# COVID-19 in Malaysia: Crucial measures in critical times

**DOI:** 10.7189/jogh.10.020333

**Published:** 2020-12

**Authors:** Kurubaran Ganasegeran, Alan Swee Hock Ch’ng, Irene Looi

**Affiliations:** 1 Clinical Research Center, Seberang Jaya Hospital, Ministry of Health Malaysia, Penang, Malaysia; 2 Medical Department, Seberang Jaya Hospital, Penang, Malaysia

Malaysia contracted a high number of COVID-19 positive cases among the Southeast Asian countries. Albeit the global COVID-19 pandemic trend is increasing, Malaysia is seeing a decrease on the number of infections, with high recoveries and low mortality rates [1]. This viewpoint aims to discuss the targeted containment strategies executed by Malaysia, which till date is showing positive responses in controlling the spread of COVID-19.

## SITUATIONAL ANALYSIS

**Figure 1:**
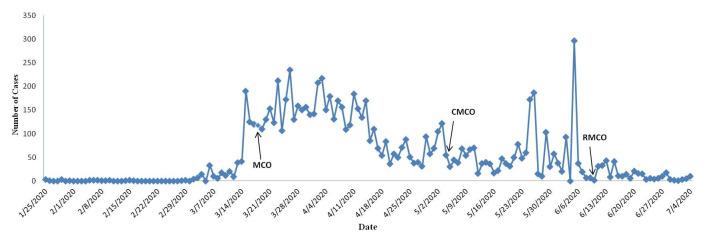
Trends of COVID-19 positive cases over time in Malaysia. Movement Control Order (MCO) was enacted on March 18, 2020 with full enforcement of legislative and non-pharmacological measures. Conditional Movement Control Order (CMCO) was enforced on May 4, 2020 with soft-opening of the economic and business sector that is subjected to strict Standard Operating Procedures (SOPs) and high compliance to non-pharmacological measures. Recovery Movement Control Order (RMCO) was introduced from June 10, 2020 with gradual opening of social, religious, business, sports and educational activities to resume under strict SOPs and high compliance to non-pharmacological measures, with the exception that international borders remained closed. Non-pharmacological measures include the avoidance of 3Cs and the practice of 3W’s. Data was available till July 04, 2020 [1,3].

As of July 04, 2020, Malaysia recorded 8658 COVID-19 positive cases, with 121 deaths and 8461 recoveries, leaving with only 76 active cases [1]. The first wave of the outbreak (January 25, 2020 - February 16, 2020) reported 22 cases and constituted mostly of imported cases [2]. Malaysia noted zero new cases until February 27, 2020, however, beyond this date marked the beginning of the second wave that observed an exponential rise of daily positive cases. As of April 10, 2020, Malaysia recorded 4346 positive cases with 70 deaths, being one of the highest across the Southeast Asian countries [2]. **Figure 1** shows time trends of positive COVID-19 cases overtime, since the first day when Malaysia contracted a positive case up to July 04, 2020. The figure shows a declining trend with the implementation of the four phase (reviewed every two weeks apart in view of COVID-19 incubation period of fourteen days) Movement Control Order (MCO) that was enacted since March 18, 2020. With declining trends, Malaysia further enforced the Conditional Movement Control Order (CMCO) and the Recovery Movement Control Order (RMCO) that have seen cases declining to single digits per day. However, periodic peaks were observed during the CMCO phase due to detection of positive cases among migrants in detention centers (**Figure 1**) [1].

## CRUCIAL PUBLIC HEALTH MEASURES IN MALAYSIA

Measures to contain the outbreak were formulated based on three principal strategies: (1) slowing introduction of global infections; (2) slowing infection of local outbreaks; and (3) executing community mitigation strategies. Travel restrictions for citizens, suspension of immigration facilities to travelers from affected regions, isolation of confirmed cases and quarantine of exposed individuals were imposed to slow introduction of global infections [4,5]. Entry and exit points were monitored closely with thermal scans installed to screen potential infectees [5].

An exponential growth of COVID-19 cases was triggered in the second wave (from February 27, 2020) due to a massive cluster gathering within the state of Selangor [2]. The Movement Control Order (MCO) that promulgated restriction of movement and activities under the Prevention and Control of Infectious Diseases Act 1988 and the Police Act 1967 was enforced on March 18, 2020 [6]. Under MCO, the following measures were implemented: (1) prohibition of mass movements, religious, sports, social and cultural activities; (2) closure of business premises except for daily necessities and needs services; (3) self-quarantine and health check measures for those who returned from abroad; (4) restrictions on tourists and visitors; (5) closure of schools, kindergartens and higher institutions of learning; and (6) closure of all government and private premises except for essential services like water, electricity, telecommunications, etc [6]. Spatial hotspots (red zones) with high incidence of positive infections (≥40 cases) would trigger an Enhanced Movement Control Order (EMCO) (a full lockdown) [7]. Such measures slowed local infections and enabled health care authorities to perform contact tracing, identify potential clusters for investigation and optimize health care resources for screening and treatment. Violators of the MCO regulations are subjected to penalties under the Penal Code, which is under a federal gazette; where violators may receive a penalty of up to MYR 1000 (approximately US$ 230 at the time of writing) and/or up to six months imprisonment [1].

With legislative and non-pharmacological interventions being put into place, the success of compliance was highly correlated with community engagement and support. The implemented interventions were concurrently executed with community mitigation strategies. Key community mitigation measures include: (1) obeying the cancellation or postponements of ad hoc or planned events, sports and religious activities; (2) high compliance on the practice of physical distancing measures and the usage of face mask; (3) reducing flight and public transportation services; (4) self-quarantine at home; (5) changes to crucial essential services like funerals to minimize crowd size and exposure to body fluids; and (6) avoidance of misinformation – verified and clear information regarding COVID-19 needs to be delivered on-time and consistently to the public to avoid fake news, rumors and panic.

Conveying accurate and rapid information on COVID-19 risk factors, transmissibility, clinical features and prevention strategies via social, print or electronic media would enhance community’s understanding of the disease. Consistent daily interaction between the government and the people to digest latest statistical updates and the do’s and don’ts during MCO via Short Messaging Service (SMS) reminders, daily press conference and social media posts had maintained transparency and compliance for successful execution of legislative measures [1]. The level of public commitment was appreciable when the Malaysian government announced alternatives to the conventional bazaars during the fasting month with novel alternatives; such as to use food delivery services and e-services. This prevented massive gatherings that would increase the risk of infections.

With declining trend of positive cases, Malaysia executed a relaxed Conditional Movement Control Order (CMCO) that aims to carefully re-open the country’s major economy in phases [1]. Malaysia adopted the carefully crafted Standard Operating Procedures (SOPs) based on the avoidance of 3Cs (Crowded places, Confined spaces, Close conversation) and the practice of 3Ws (Wash hands, Wear masks, Warn against risks, symptoms, prevention and treatment) during the enforcement of the four main legislation acts through the MCO, EMCO, CMCO and RMCO till date, which have seen success in flattening the epidemic curve [1].

## CRITICAL PUBLIC HEALTH IMPLICATIONS AND FUTURE DIRECTION

Contact tracing is more critical when individuals with high risk of transmission within detected clusters are difficult to track rapidly. Other vulnerable groups such as migrants or homeless people with poor living conditions compounded with overcrowding have increased susceptibility towards the spread of potential local contagion [8]. The execution of institutional quarantining of people who have been in contact with confirmed or probable cases may be unrealistic at certain times as this overwhelms the system and may lead to more infections. Self-quarantine may be a more realistic measure [9]. Rigorous and routine screening efforts and disinfection process in public or overcrowded places is important to control the spread of the infection. As severe complications and high mortality rates is correlated with older people [1], more focused and targeted interventions of infection control efforts and potential treatment modalities should be prioritized to the aged population. Specific and more comprehensive guidelines on hygienic processes and infection control strategies should be directed to elderly care homes. While there are guidelines in place [10], monitoring the compliance in these homes are crucial to prevent the spread of COVID-19 among the elderly.

Lockdowns have unprecedented psychological repercussions to people [11]. To overcome stress, anxiety, depression or isolation, Malaysia has introduced psychological services through the establishment of care lines and virtual counseling sessions [12]. Misinformation cause panic, as such, it is crucial to provide verified information of the contagion through daily updates.

## CONCLUSIONS

We are in the midst of battling the transmission of a pathogen that disrespects national and international borders with many uncertainties. While being the most potent infection to the human population globally, the anticipated hazards and implications are expected to prolong for months to come until appropriate evidenced based treatment modalities and vaccines are discovered. Pending answers to the many puzzles of COVID-19 from the scientific and medical perspective, it is crucial to reckon the importance of executed public health measures in such critical times to disallow an uncontrolled spread and replication of the virus to the human living environment, which would ultimately be difficult to tackle. Malaysia has taken a unique targeted approach in controlling the COVID-19 outbreak.

**Figure:**
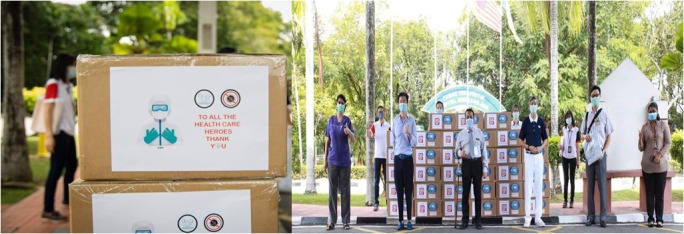
Photo: Support to governmental health facilities and health care workers who advocate standard operating procedures such as movement restriction, social distancing and face masking to the public during Movement Control Order (MCO) in Malaysia (photo collection by the authors).
